# Expression, Characterization and Synergistic Interactions of *Myxobacter* Sp. AL-1 Cel9 and Cel48 Glycosyl Hydrolases

**DOI:** 10.3390/ijms9030247

**Published:** 2008-02-29

**Authors:** Norma Ramírez-Ramírez, Eliel R. Romero-García, Vianney C. Calderón, Claudia I. Avitia, Alfredo Téllez-Valencia, Mario Pedraza-Reyes

**Affiliations:** 1 Instituto de Investigación en Biología Experimental (IIBE), Facultad de Química, Universidad de Guanajuato. P.O. Box 187. Guanajuato, Gto. 36050, Mexico.; 2 Instituto de Ciencias de la Salud, Universidad Autónoma del Estado de Hidalgo. Abasolo 600, Pachuca, Hgo. 42000, Mexico

**Keywords:** Cellulose, Cel9, Cel48 cellulases, Synergism, *Myxobacter* Sp. AL-1

## Abstract

The soil microorganism *Myxobacter* Sp. AL-1 regulates in a differential manner the production of five extracellular cellulases during its life cycle. The nucleotide sequence of a *cel9-cel48* cluster from the genome of this microorganism was recently obtained. *Cel48* was expressed in *Escherichia coli* to generate a His_6_-Cel48 protein and the biochemical properties of the pure protein were determined. Cel48 was more efficient in degrading acid-swollen avicel (ASC) than carboxymethylcellulose (CMC). On the other hand, *cel9* was expressed in *Bacillus subtilis* from an IPTG-inducible promoter. Zymogram analysis showed that after IPTG-induction, Cel9 existed in both the cell fraction and the culture medium of *B. subtilis* and the secreted protein was purified to homogeneity by FPLC-ionic exchange chromatography. The exocellobiohydrolase Cel48 showed a synergism of 1.68 times with the endocellulase Cel9 during ASC degradation using an 8.1-fold excess of Cel48 over Cel9. Western blot analysis revealed that both proteins were synthesized and secreted to the culture medium of *Myxobacter* Sp. AL-1. These results show that the *cel9-cel48* cluster encodes functional endo- and exo-acting cellulases that allows *Myobacter* Sp. AL-1 to hydrolyse cellulose.

## 1. Introduction

*Myxobacter* Sp. AL-1 regulates in a temporal fashion the production of at least five extracellular cellulases with different molecular weights. A 29 kDa protein, part of this enzyme battery, was purified to homogeneity and showed to possess chitosanase-cellulase activity [1]. Furthermore, a gene encoding a family 9 extracellular cellulase was cloned from the genome of *Myxobacter* Sp. AL-1 [2]. Sequences located downstream of *cel9* showed the existence of a contiguous partial reading frame, which putatively coded for another cellulase, termed cel48 [2]. The first gene of this cluster, *cel9*, was successfully expressed in *Escherichia coli* and the biochemical properties of the pure protein were very similar as those exhibited by related cellulases produced by thermophilic bacteria [2]. Moreover, results of a Gapped-blast alignment analysis revealed that Cel9 is a modular enzyme composed of N-terminal catalytic and C-terminal type IIIc cellulose binding domains, respectively [3].

The complete degradation of cellulose occurs with the action of endo-1,4-β-glucanases and exo-1,4-β-glucanases. The endo-acting enzymes attack the cellulose chain internally whereas the exo-1,4-β-glucanases attack the reducing or non-reducing end of the cellulose chain to generate cellobiose or glucose [4, 5]. The ability of *Myxobacter* Sp. AL-1 to grow in CMC as a sole carbon source (Our unpublished results) and the presence in this microorganism of a *cel9-cel48* cluster suggest that this soil microorganism possesses the enzymatic machinery to degrade cellulose. However, since the full nucleotide sequence of *cel48* was not available, the biochemical properties of its encoding product remained unknown. In this communication, we report the expression of *cel48* in *E. coli* and the purification of its encoding product as a His_6_-Cel48 protein. His_6_-Cel48 showed to possess biochemical properties as those described for cellobiohydrolases from bacterial origin belonging to the family 48 of the glycosyl hydrolases. Moreover, the expression of *cel9* from an IPTG-inducible promoter was carried out in *Bacillus subtilis*. The recombinant Cel9 protein was secreted to the culture medium of *B. subtilis* cells and was purified to homogeneity by FPLC. Results from a Western-blot analysis revealed that Cel48 as well as Cel9 are produced and secreted by *Myxobacter* Sp. AL-1; moreover, both proteins acted together, in a synergistic manner, to process the degradation of acid-swollen avicel.

## 2. Methodology

### Bacterial strains, plasmids and growth conditions

Strains used in this study are shown in Table 1. Media used was Luria-Bertani (LB) [6]. When necessary antibiotics were added to media at the following final concentrations: ampicillin (Amp), 100 μg/mL; kanamycin (Kan), 10 μg/mL. Cells were grown in liquid media with vigorous aeration or on solid media at 37°C. Growth in the cultures was determined by measuring the optical density at 600 nm using an Ultrospec Pharmacia spectrophotometer.

### Genetic and molecular biology techniques

Transformation of *E. coli*, small and large scale preparation of plasmid DNA, enzymatic manipulations and agarose gel electrophoresis were performed by standard techniques [7]. PCR products for nucleic acid sequencing were obtained with homologous oligonucleotides and Vent DNA polymerase (New England Biolabs, Beverly, MA).

### Plasmid construct to overexpress cel48 and generate a His_6_-Cel48 protein

The open reading frame of *cel48* was amplified by PCR, using plasmid pPERM123 (Table 1) with specific oligonucleotide primers designed to insert *Bam*HI and *Sal*I restriction sites into the cloned DNA. The PCR fragment was first introduced into PCR-Blunt-II-TOPO plasmid to generate pPERM400 which was replicated into *E. coli* XL-10 Gold Kan^R^ (Stratagene, La Jolla, CA). pPERM400 was digested with *Sal*I and *Bam*HI and the *cel48* ORF fragment was inserted in-frame into the *Bam*HI/*Sal*I site of the expression vector pQE30 (QIAGEN Inc. Valencia, CA); the resulting construction pPERM407 was introduced into *E. coli* XL-10 Gold Kan^R^ (Stratagene, La Jolla, CA) generating strain *E. coli* PERM407. The proper in-frame insertion of the *cel48* fragment was assessed by both restriction analysis and DNA sequencing.

### Purification of His_6_-Cel48 and Cel9

*E. coli* PERM407 was grown in 50 ml of LB medium supplemented with Amp to an OD_600nm_ of 0.5. Expression of the *cel48* gene was induced during 2 h, at 37°C by addition of isopropyl-β-D-thio-galactopyranoside (IPTG) to 0.2 mM. Cells were collected by centrifugation and washed two times with 50 mM Tris-HCl (pH 6.0, 10 mL), 150 mM NaCl (buffer A). The cells were disrupted in buffer A (10 mL) containing lysozyme (5 mg/mL) for 1 h at 37°C. The cell homogenate was subjected to centrifugation (27,200 × g) to eliminate undisrupted cells and cell debris and the supernatant was applied to a 5 mL Ni-NTA-Agarose (QIAGEN Inc.) column, previously equilibrated with buffer A. The column was washed with buffer A (50 mL) and buffer A containing 10 mM imidazole (50 mL) and the protein bound to the resin was eluted with buffer A (6 mL) containing imidazole (200 mM); 4 mL fractions were collected during this last step. Purified fractions were analyzed by sodium dodecyl sulfate polyacrylamide gel electrophoresis (SDS-PAGE) as previously described [8].

The protein secreted to the culture medium by *B. subtilis* PERM272 after 12 h of IPTG induction was precipitated with ammonium sulfate and the lyophilized precipitate was subjected to purification on a Mono Q HR 16/10 anion-exchange column as previously described [2].

### Immunological procedures

Proteins present in the culture medium of *B. subtilis* PERM272 and cell extract from *E. coli* PERM407 were separated in SDS 10% polyacrylamide gels. The proteins bands corresponding to Cel9 and His_6_-Cel48, respectively, were cut out from the gel, homogenized, mixed 1:1 with Freud adjuvant, and injected into New Zealand White rabbits. The antiserums were collected 12 weeks after the first immunization. Western blot analyses were performed with the antiserums diluted 10,000-fold and then processed with an ECL (Enhanced chemiluminescence) western blotting analysis system (Amersham Pharmacia, UK).

### Enzyme and protein assays

Cellulase activity was determined by measuring the enzymatic release of reducing sugars as previously described [9, 10]. Acid-swollen avicel (ASC) and CMC were used at a final concentration of 0.5% and 1%, respectively. *p*-Nitrophenyl-β-D-cellobioside (*p*-NP-Glc_2_) and *p*-nitrophenyl-β-D-cellotrioside (*p*-NP-Glc_3_) were used at a final concentration of 4 mM. Units of enzyme activity were reported as nmols of reducing sugars released/min/mg of protein. ASC was prepared as previously described [11].

### SDS-PAGE and cellulase zymography

Electrophoresis in SDS-polyacrylamide gels was carried out as previously described by Laemmli [8]. Gels were stained with Coomassie blue R-250. Zymograms of cellulase activity were performed in SDS-polyacrylamide gels containing CM-cellulose according to a previously described protocol [2].

## 3. Results and Discussion

**Figure 1.**
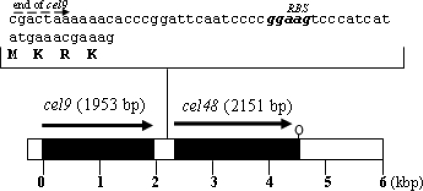
**Genetic organization of the *cel9-cel48* cluster of *Myxobacter* Sp. AL. 1.** Solid arrows show the ORFs of *cel9* and *cel48*, respectively. A steam loop structure downstream of *cel48* (white) represent a putative transcriptional terminator. The end of *cel9* is shown with a discontinuous arrow. The presumptive ribosome binding site of *cel48* is shown in bold italics.

Since regions lying downstream of *cel9* showed the presence of a second partial ORF, encoding a putative cellulase, it was of further interest to characterize this gene. Thus, the full DNA sequence of this second gene was obtained by PCR walking using homologous oligonucleotides and the results revealed an ORF of 2151 bp (GenBank accession number 778327) encoding a protein with a predicted molecular mass of 81 kDa and a theoretical isoelectric point of 5.4 (Figure 1). A Gapped-blast analysis (results not shown) showed that this protein possessed identity with cellobiohydrolases of bacterial origin grouped in the family 48 of the glycosyl hydrolases [12], therefore this gene was termed *cel48*. Interestingly, Cel48C a 118 kDa cellobiohydrolase from *Paenibacillus* sp. BP-23 [13] contains a type 3a cellulose-binding module as well as a fibronectin-III domain on its C-terminal end which are absent in Cel48.

**Figure 2.**
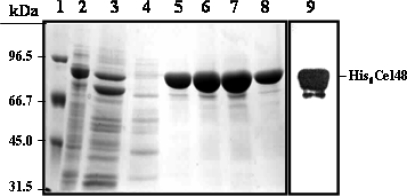
**SDS-PAGE analysis of His_6_-Cel48 purification through a Ni-NTA-agarose column.** Expression and purification of His_6_-Cel48 from *E. coli* PERM407 was performed as described in Methodology. (Lane 1, molecular weight markers; Lane 2, *E. coli* PERM407 lysate; Lane 3, flow through; Lane 4, protein fraction eluded with 10 mM imidazol; Lanes 5–8, fractions eluted from the column with 200 mM imidazol; Lane 9, ~100 μg of protein from an *E. coli* PERM407 lysate were separated on a SDS–12% polyacrylamide gel and transferred to nitrocellulose membranes. The blot was probed with a polyclonal anti-Cel48 rabbit antibody which was diluted 10,000-fold and then processed with an ECL Western-blotting analysis system.)

To determine the biochemical and biophysical properties of Cel48, this protein was produced and purified from *E. coli* cells transformed with plasmid pPERM407. Cell-free extracts of the transformed cells showed the presence of a protein of around 81 kDa (Figure 2, Lane 2), which was subsequently purified on a Ni-NTA-agarose (Figure 2, Lanes 5–8) and was recognized by an anti-Cel48 antibody in the cell free extracts of this *E. coli* strain (Figure 2, Lane 9).

Although the recombinant His_6_-Cel48 protein catalyzed the hydrolysis of ASC and CMC, the enzyme was more active against the former substrate (Table 2). It has been previously described that family 48 cellobiohydrolases catalyze the hydrolysis of cellulosic substrates such as ASC in a slow manner [9, 13]. In agreement with this observation, maximum hydrolysis of ASC and CMC by His_6_-Cel48 occurred after 18–24 h at pH 6 (Results not shown).

Moreover, the recombinant enzyme showed to be very stable at least for 2 h periods, at 23, 37 and 45°C; however, the enzyme was rapidly inactivated at 60°C or higher temperatures (Results not shown). On the other hand, as shown in Table 2, no activity of His _6_-Cel48 was detected against avicel and the oligomeric substrates *p*-nitrophenyl β-D-cellobioside (*p*-NP-Glc_2_) and *p*-nitrophenyl β-D-cellotrioside (*p*-NP-Glc_3_). Thus, in terms of substrate specificity, His_6_-Cel48 resembled the catalytic domain of Cel48A from the thermophile bacterium *Thermomonospura fusca* (Cel48Acd), since the recombinant form of this enzyme showed activity against ASC and CMC but was inactive against cello-oligosaccharides [9]. Other properties such as optimal pH and thermal stability were also shared by both enzymes.

The synergistic degradation of cellulose by endoglucanases and exoglucanases has been previously documented [13–17]. It has been proposed that the synergistic reaction between both classes of enzymes involves a mechanism in which the endoglucanases attack the amorphous portions of cellulose followed by the action of exoglucanases which release cellobiose from the internal nicks [14].

**Figure 3.**
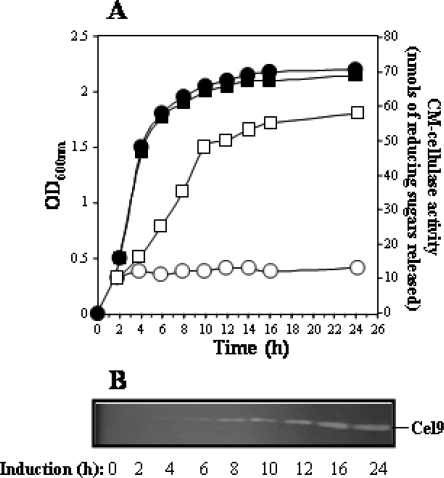
**Analysis of *cel9* induction in a *B. subtilis* strain harboring a P*spac-cel9* construction. A.** *B. subtilis* PERM272 was grown at 37°C in LB medium to an OD_600nm_ of 0.5. At this point the culture was divided in two equal subcultures; one of the subcultures was left as a control (●, ○) and the other was supplemented with 2 mM IPTG (■, □). Aliquots (1 mL) were taken at different times from both subcultures; cells were separated by centrifugation and the supernatant used to determine CM-cellulase activity (○, □) as described in Methodology. (●, ■) OD_600nm_. B. Samples (~100 μg protein) collected at different times from the supernatant of the IPTG-induced subculture were analyzed on zymograms as described in Methodology.

The existence of contiguous *cel9* and *cel48* genes encoding endo- and exo-acting cellulases suggested that *Myxobacter* Sp. AL-1 possessed the enzymatic machinery to degrade cellulose. To investigate whether Cel48 together with Cel9 catalyzed the degradation of ASC in a synergistic manner it was necessary to purify Cel9. To this end, the protease and β-glucanase deficient strain *B. subtilis* 1A751 (Table 1) was used as a host to express *cel9*. Thus, *cel9* was cloned in the plasmid pDG148 [18] under the control of the IPTG-inducible P*spac* promoter and the resulting construction was introduced in *B. subtilis* 1A751 generating the strain *B. subtilis* PERM272. As shown in Figure 3, addition of IPTG to a mid logarithmic phase culture of strain *B. subtilis* PERM272 induced around five times the levels of CM-cellulase expression. The maximum levels of CM-cellulase activity were reached 10 h after addition of the inducer and these values were maintained during the following 14 h. SDS-PAGE and zymogram analysis revealed the presence of a protein with the predicted molecular mass of Cel9 that also possessed CM-cellulase activity in the culture medium of *B. subtilis* PERM272.

**Figure 4.**
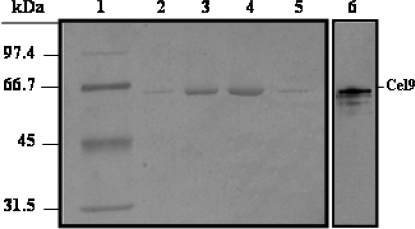
SDS-PAGE analysis of Cel9 purification through a Mono Q HR 16/10 column. Fractions (15 μL) from the peak of CM-cellulase activity from a Mono Q HR 16/10 anion-exchange column (Lanes 2–5) were analyzed on a SDS-12% polyacrylamide gel. (Lane 1, Molecular weight markers. Lane 6, ~100 μg of protein from the supernatant of an IPTG-induced *B. subtilis* PERM272 culture were separated on a SDS–12% polyacrylamide gel and transferred to a nitrocellulose membrane. The blot was probed with a polyclonal anti-Cel9 rabbit antibody which was diluted 10,000-fold and then processed with an ECL Western-blotting analysis system.)

Since *B. subtilis* was able to synthesize and secrete Cel9 we used a culture of strain *B. subtilis* PERM272 induced with IPTG for 12 h as a source to purify this protein. To this end, after separating the cells, the secreted proteins were concentrated by ammonium sulfate precipitation and then subjected to purification on a Mono-Q HR 16/10 anion-exchange column. After applying a linear salt gradient, Cel9 was solved on this column as a single peak of CM-cellulase activity which was eluded with around 0.12 M NaCl (Results not shown). SDS-PAGE analysis showed that Cel9 was purified to apparent homogeneity after this chromatographic step (Fig. 4). Following this protocol, Cel9 was purified five times with a 60% recovery (Results not shown). Results shown in Figure 4 (Lane 6) revealed that the ~66 kDa purified protein was specifically recognized by an anti-Cel9 antibody in the supernatant of the IPTG-induced strain *B. subtilis* PERM272.

**Figure 5.**
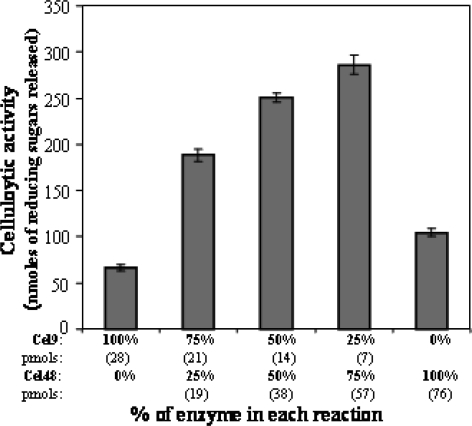
Cellulase activity (nmols of reducing sugars released) against ASC by mixtures of Cel9 and Cel48 after 18 h of incubation at 40°C. Purified Cel9 and Cel48 were used alone or combined at the indicated ratios in the assay mixtures (500 μL). The pmols of each enzyme used in the assay mixtures are indicated in parenthesis.

Having available purified samples of both Cel9 and Cel48 it was further investigated whether these proteins catalyzed the degradation of ASC in a synergistic manner. To this end, either individual samples or different ratio’s mixtures of Cel9 and Cel48 were incubated with ASC at a final concentration of 0.5% during 18 h at 40°C. A synergistic interaction between Cel9 and Cel48 during ASC degradation was observed since the activity of all mixtures were significantly higher than that of either enzyme assayed alone (Fig. 5). The enzyme Cel48 showed a synergism of 1.68 times with the endoglucanase Cel9 during ASC degradation using an 8.1-fold excess of Cel48 over Cel9 (Figure 5).

**Figure 6.**
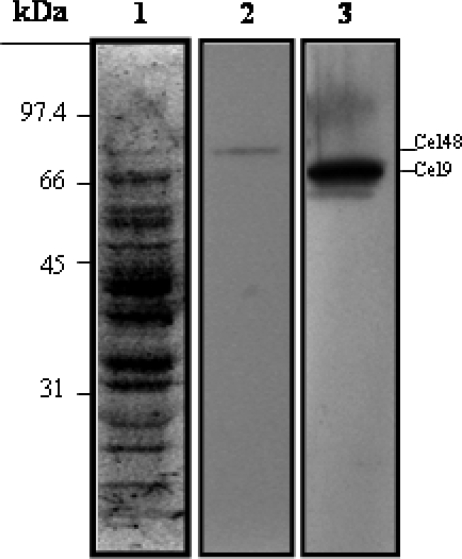
Westen blot analysis of the synthesis of Cel9 and Cel48 during *Myxobacter* Sp. AL-1 growth. 100 μg of protein from the culture medium collected at 24 h were separated by SDS–PAGE and transferred to nitrocellulose membranes. The blots were stainned with Ponceau’s red (Lanes 1) and probed with polyclonal anti-Cel48 (Lane 2) or anti-Cel9 (Lane 3) rabbit antibodies which were diluted 10,000-fold and then processed with an ECL Western-blotting analysis system.

Taken these results together is reasonable to conclude that the genetic information present in the *cel9-cel48* cluster of *Myxobacter* Sp. AL-1 encodes functional enzymes which catalyze synergistically the degradation of cellulose. However, as noted above, both, Cel9 and Cel48 were synthesized in heterologous hosts and the existence of these enzymes in *Myxobacter* Sp. AL-1 remained to be demonstrated. Regarding this issue, previous results of a zymogram analysis suggested that a ~70 kDa protein secreted into the culture medium of *Myxobacter* Sp. AL-1 could correspond to Cel9 [2]. However, to date the production of Cel48 by *Myxobacter* Sp. AL-1 has not been proven. Thus, the polyclonal antibodies generated against Cel9 and Cel48 were used to localize both proteins in the cell culture of this microorganism. The results shown in Figure 6 reveled that the anti-Cel9 and anti-Cel48 antibodies were able to recognize proteins with molecular masses of around 67 and 82 kDa, respectively, in the supernatant of a 24 h culture of *Myxobacter* Sp. AL-1. These results strongly suggest that Cel9 and Cel48 are produced and secreted by *Myxobacter* Sp. AL-1 to process the hydrolysis of cellulose.

In conclusion, the results described in this work add new evidence on the existence of the enzymatic machinery to process the synergistic degradation of cellulose in the soil microorganism *Myxobacter* Sp. AL-1.

## Figures and Tables

**Table 1.** Bacterial strains used in this study.

Bacterial strain	Genotype and description	Reference or source
*E. coli* XL10-Gold Kan^R^	{Tet^r^ Δ(*mcrA*) *183*, Δ(*mcrBC-hsd SMR-mrr*); Kan *173 endA1 sup E44 thi-1 recA1 gyrA96 relA1 lacHte* [F’ *proAB lacI*^*q*^*Z*DM15 Tn*10* (Tet^r^) Tn5 (Kan^r^) Amy]}	(Stratagene, La Jolla, CA.)
*E. coli* DH5α	{*deoR* [φ80d*lac*Δ(*lacZ*)M15] *recA*1 *endA*1 *gyrA*96 *hsdR*17 (rk^−^, mk^+^) *phoA supE*44 *thi*-1 *relA*1 Δ(*lacI*ZYA-*argF*)U169}.	Laboratory stock
*E. coli* PERM123	*E. coli* DH5α containing plasmid pPERM123 (pBR322 with a 6 kb-*EcoR*I-*EcoR*I *cel9-cel48* containing fragment).	[2]
*B. subtilis* 1A751	*egl*SΔ102, *bgl*T/*bgl*SΔEV, *npr*, *aprE*, *his*	BGSC^a^
*B. subtilis* PERM272	*B. subtilis* IA751 containing plasmid pDG148 with An IPTG-inducible P*spac-cel9* construction	[2]
*E. coli* PERM407	*E. coli* XL10-Gold containing plasmid pQE30 with an IPTG inducible PT_*5*_-*His*_*6*_-*cel48* construction	This study

a BGSC (*Bacillus* genetic stock center)

**Table 2.** Substrate specificity of recombinant His_6_-Cel48.

Substrate	Specific Activity
ASC	10.5^a^
CMC	2.75
Avicel	ND^b^
*p*-NP-Glc_2_	ND
*p*-NP-Glc_3_	ND

a Individual values are the mean of triplicate assays, where values did not deviate by more than 15% of the mean;

b ND, not detected
